# Sex Differences in the Effects of a Kappa Opioid Receptor Antagonist in the Forced Swim Test

**DOI:** 10.3389/fphar.2018.00093

**Published:** 2018-02-14

**Authors:** Abigail Laman-Maharg, Alexia V. Williams, Mikaela D. Zufelt, Vanessa A. Minie, Stephanie Ramos-Maciel, Rebecca Hao, Evelyn Ordoñes Sanchez, Tiffany Copeland, Jill L. Silverman, Angelina Leigh, Rodney Snyder, F. Ivy Carroll, Timothy R. Fennell, Brian C. Trainor

**Affiliations:** ^1^Neuroscience Graduate Group, University of California, Davis, Davis, CA, United States; ^2^Department of Psychology, University of California, Davis, Davis, CA, United States; ^3^MIND Institute, School of Medicine, University of California, Davis, Davis, CA, United States; ^4^Research Triangle Institute, Durham, NC, United States

**Keywords:** kappa opioid receptors, sex differences, antidepressants, stress, nucleus accumbens

## Abstract

There is growing evidence that kappa opioid receptor (KOR) antagonists could be a useful class of therapeutics for treating depression and anxiety. However, the overwhelming majority of preclinical investigations examining the behavioral effects of KOR antagonists have been in male rodents. Here, we examined the effects of the long-acting KOR antagonist nor-binaltophimine (norBNI) on immobility in the forced swim test in males and females of two different rodent species (C57Bl/6J and California mice). Consistent with previous reports, norBNI (10 mg/kg) decreased immobility in the forced swim test for male C57Bl/6J and California mice. Surprisingly, dose–response studies in female C57Bl/6J and California mice showed that norBNI did not reduce immobility. Pharmacokinetic analyses showed that metabolism and brain concentrations of norBNI were similar in male and female C57Bl/6J. In the nucleus accumbens of male but not female C57Bl/6J, norBNI increased phosphorylation of c-Jun N-terminal kinase (pJNK), a putative mechanism for norBNI action. However, no differences in pJNK were observed in male or female California mice. Together, these results suggest that immobility in the forced swim test is less dependent on endogenous KOR signaling in female rodents and highlight the importance of examining the effects of possible therapeutic agents in both males and females.

## Introduction

Psychosocial stress exposure is an important risk factor for the development of depression and anxiety disorders (Bruce, 2002). Kappa opioid receptors (KORs) have generated interest as a potential pharmacological target because KORs are activated by psychosocial stress (Knoll and Carlezon, 2010) and pharmacological activation of KORs induces aversion, dysphoria, and depression-like behavior in humans (Pfeiffer et al., 1986; Walsh et al., 2001) and animals (Todtenkopf et al., 2004; Carlezon et al., 2006; Land et al., 2008). In addition, KOR antagonists have been reported to have anxiolytic or antidepressive relevant properties in a variety of behavioral tests measuring affect, including the forced swim (Pliakas et al., 2001; Mague et al., 2003; Beardsley et al., 2005; Chartoff et al., 2012), social interaction (Bruchas et al., 2011), and elevated plus maze (Knoll et al., 2007; Peters et al., 2011) assays. However, there are two major gaps in the literature. First, the overwhelming majority of preclinical studies on KOR antagonists have been conducted in male rodents (but see, Russell et al., 2014), so it is unclear whether these antagonists have similar properties in females. This is an essential question because women are almost twice as likely to develop depression as men (Kessler, 2003), and sex differences in physiological responses to stress may affect risk for depression (Bale and Epperson, 2015; Laman-Maharg et al., 2017). Second, most studies have focused on the short-term effects of KOR antagonists in stressful contexts. However, there is growing evidence that exposure to stressful conditions such as social defeat induces long-term changes in the behavioral effects of KOR (Kudryavtseva et al., 2004a,b; Donahue et al., 2015; Polter et al., 2017).

A common assay for identifying pharmaceuticals with antidepressant properties in preclinical settings is the forced swim test (Porsolt et al., 1977; Castagne et al., 2011). In male C57Bl/6J (Falcon et al., 2015) mice, the long-acting KOR antagonist nor-binaltophimine (norBNI) (Horan et al., 1992) has been found to reduce immobility in a single 6 min forced swim. Modified versions of the forced swim test have used swim trials on two consecutive days (Cryan et al., 2005). In this 2-day test, norBNI reduces immobility in male rats (Pliakas et al., 2001; Mague et al., 2003; Beardsley et al., 2005). One modified form uses repeated 5 min bouts of forced swim on day 2, and in this protocol, the selective KOR antagonist norBNI decreases immobility (McLaughlin et al., 2003). This 2-day protocol has been found to induce other KOR-dependent behavioral (Land et al., 2008) and neurobiological (Bruchas et al., 2007a) responses. Interestingly, social defeat stress induces KOR activation (McLaughlin et al., 2006) and has been reported to increase time spent immobile in some cases (Jin et al., 2015) but not others (Krishnan et al., 2007). There is growing evidence that over a period of days or weeks, stressors such as defeat reduce the efficacy of KOR on behavior (Al-Hasani et al., 2013; Laman-Maharg et al., 2017). Overall, while there is strong evidence that KOR antagonists have antidepressant-like effects in the force swim, the extent to which this effect generalizes to females or to females that have experienced stress is unclear.

Here, we tested the behavioral effects of the long-acting KOR antagonist norBNI in males and females of two species of rodent. Experiments were conducted in female California mice (*Peromyscus californicus*), because the social system of this species allows for social defeat stress to be studied in both males and females (Trainor et al., 2011, 2013). When unexpected results were observed in California mice, we replicated a subset of these studies in C57Bl/6J to determine whether there are species differences in the behavioral effects of norBNI. In addition to behavioral outcome measures, we assessed two mechanisms that could potentially explain sex differences in KOR antagonist action. First, we examined the pharmacokinetics of norBNI in the brain and plasma of both males and females. Second, we examined the effects of norBNI on the phosphorylation of c-Jun N-terminal kinase (pJNK) in the nucleus accumbens (NAc). pJNK is thought to be an important mechanism of norBNI action in the forced swim test (Bruchas et al., 2007b).

## Materials and Methods

### Experiments

#### Experiment 1: Effects of norBNI and U50,488 on Immobility in Female California Mice

**FIGURE 1:**
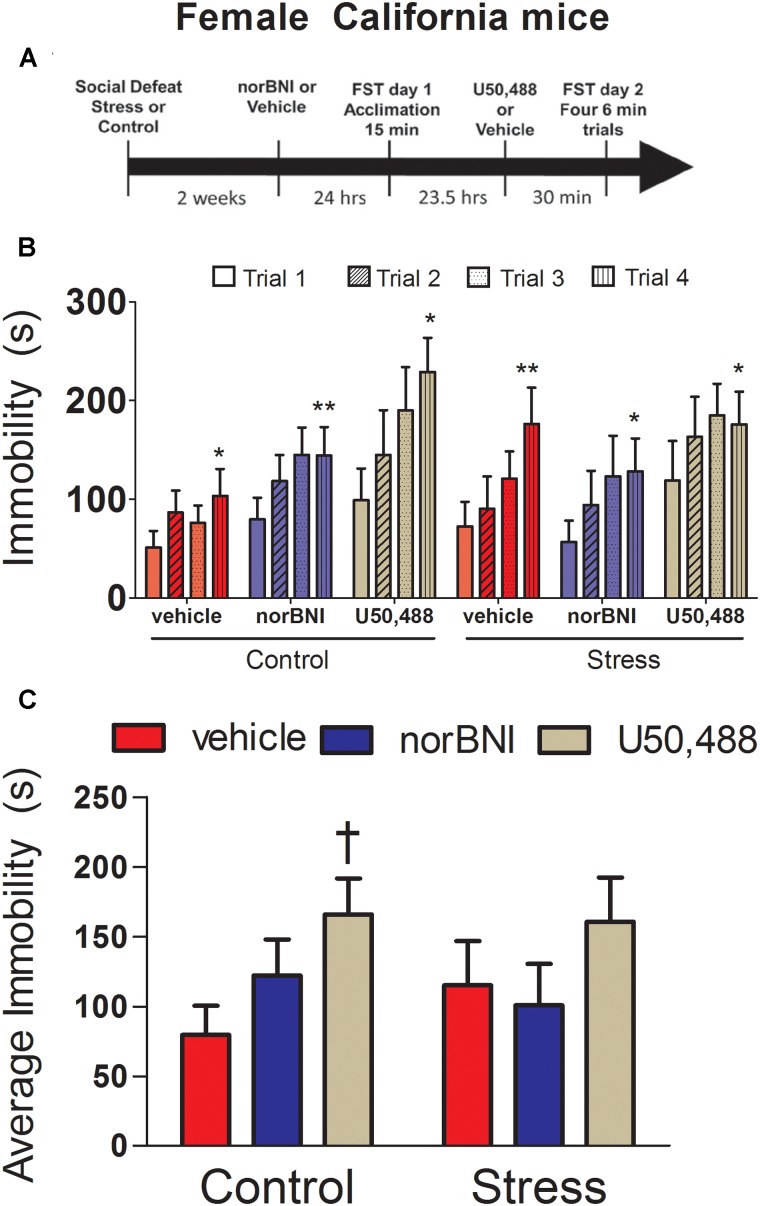
Effects of 10 mg/kg nor-binaltophimine (norBNI) and 10 mg/kg U50,488 on immobility in female California mice. **(A)** Experimental timeline of analyses. **(B)** Day 2 immobility for the four trials for control and stressed female California mice. **(C)** Average immobility across the four Day 2 trials. Immobility scores were analyzed with two-way repeated measures ANOVA testing for stress and drug treatment. ^∗^*p* < 0.05, ^∗∗^*p* < 0.01 paired *t*-test trial 1 vs. trial 4; ^†^*p* < 0.05 planned comparison vs. vehicle, *n* = 8–12 per group. Error bars are SEM.

Female California mice were randomly assigned to either defeat or control conditions and forced swim testing was conducted 2 weeks later. Mice were injected with either norBNI or vehicle 24 h before day 1 of testing and then injected a second time with U50,488 or vehicle (for both vehicle and norBNI groups) 30 min before day 2 of testing. norBNI inhibits the mu opioid receptor (MOR) for the first 24 h of injection, but at 24 h, becomes much more potent and selective for KOR (Endoh et al., 1992). Therefore, in order to avoid behavioral effects of MOR antagonism, mice were injected with either norBNI or vehicle 24 h before day 1 of forced swim testing (**Figure 1A**). Following the last swim trial, mice were anesthetized with isoflurane and euthanized by decapitation. Brains were immediately collected, flash frozen on dry ice, and stored at -40°C. Vaginal lavage indicated that 47% of females were in proestrus or estrus on day 2 of testing.

#### Experiment 2: Effects of norBNI on Immobility in Male California Mice

Male California mice were randomly assigned to receive either vehicle [10% tween in sterile phosphate buffered saline (PBS)] or 10 mg/kg norBNI 24 h before day 1 of forced swim testing. Forced swim testing was conducted as in Experiment 1 and brains were collected immediately after the last swim trial as described above.

#### Experiment 3: Effects of norBNI on Immobility in Male and Female C57Bl/6J

Male C57Bl/6J mice were randomly assigned to receive vehicle (10% tween in sterile PBS), 10 mg/kg, or 25 mg/kg of norBNI. Female C57Bl/6J mice were randomly assigned to receive vehicle, 0.5 mg/kg norBNI, 10 mg/kg norBNI, or 25 mg/kg norBNI. In pilot studies, females did not respond to 10 mg/kg of norBNI so an extra lower dose was added to test whether females were hypersensitive to norBNI (Greenberg et al., 2014). Mice were injected with either norBNI or vehicle 24 h before day 1 of testing (**Figure 3A**). Brains were collected immediately after the last swim trial as in Experiment 1. Vaginal lavage indicated that 34% of females were in proestrus or estrus on day 2 of testing.

#### Experiment 4: Pharmacokinetics of norBNI and JDTic and Quantification of pJNK in the Nucleus Accumbens of Male and Female C57Bl/6J

Male and female C57Bl/6J mice were injected with 10 mg/kg norBNI dissolved in 10% tween 80 in sterile PBS. We chose this dose because 10 mg/kg norBNI decreased immobility in both male California and C57Bl/6J mice. Mice were randomly assigned to be euthanized at 1, 5, 48 h, or 3 weeks after receiving the drug treatment. Mice were anesthetized with isoflurane and euthanized by decapitation. Trunk blood was collected, mixed with 10 μl heparin (1000 USP/ml), and placed on wet ice. Brains were immediately collected, flash frozen on dry ice, and stored at -40°C. Tools were cleaned and dried between mice to avoid contamination. Brains were rinsed in sterile PBS immediately before being frozen on dry ice. Blood was centrifuged at 4°C for 12 min at 9800 rpm. Plasma was collected and stored at -40°C. Whole brain and plasma samples were analyzed using liquid chromatography–mass spectrometry. A separate set of male and female C57Bl/6J mice were injected i.p. with 10 mg/kg of the KOR antagonist JDTic dissolved in 10% tween 80 in sterile PBS. All procedures and timelines were exactly the same as the norBNI study.

Flash frozen brains from the male and female C57Bl/6J mice (from Experiment 3) and unstressed male and female California mice (from Experiments 1 and 2) treated with either vehicle or 10 mg/kg norBNI were sectioned at 500 μm on a cryostat. Punch samples were immediately collected from the NAc bilaterally using a 16 g (1.194 mm diameter) punch tool. Punches were stored at -40°C until used for western blotting.

### Animals and Housing Conditions

Adult male and female California mice (3–4 months old) and 8-week old male and female C57Bl/6J *Mus musculus* were studied. All mice were group housed in same-sex groups. California mice were fed Harlan Teklad 2016 (Madison, WI, United States) and C57Bl/6J were fed LabDiet 5015 Mouse Diet (St. Louis, MO, United States). All other housing conditions were the same between the two species. Animals were maintained in a temperature-controlled room (68–74°F) on a 16L-8D cycle (lights off at 1400) with *ad libitum* water and food. Polycarbonate plastic cages containing Sanichip bedding, nestlets, and envirodri were used. All procedures were approved by the UC Davis Institutional Animal Care and Use Committee (IACUC) and conformed to NIH guidelines. Social defeat stress and social interaction testing were conducted during lights out (1400–1700) under dim red light (3 lux). Forced swim testing was conducted during lights on (0900–1300).

### Social Defeat Stress

Female California mice were randomly assigned to social defeat stress or control handling for 3 consecutive days (Trainor et al., 2011, 2013). Mice assigned to social defeat were placed in the home cage of an aggressive, same-sex sexually-experienced resident mouse. The experimental mouse remained in the resident’s cage for either 7 min or 7 attacks, whichever occurred first. Control mice were introduced to a clean, empty cage for 7 min. Each experimental mouse was exposed to a different resident for each of the three episodes of defeat stress.

### Drugs

Norbinaltorphimine dihydrochloride (norBNI) and (±)-U-50488 hydrochloride (U50,488) were obtained from Tocris (Ellisville, MO, United States). JDTic was supplied by F. Ivy Carroll. NorBNI, U50,488, and JDTic were dissolved in vehicle consisting of sterile PBS with 10% Tween 80 (Fisher Scientific, Fair Lawn, NJ, United States). All drugs were administered via i.p. injection.

### Forced Swim Test

Forced swim testing consisted of a 2-day protocol, adapted from previous studies (McLaughlin et al., 2003). All swim testing took place in an opaque cylinder (25.5 cm tall × 20 cm in diameter) filled with 14 cm of 30°C water during the light phase. Each cylinder was cleaned with Quatricide (1:64, Quatricide PV in water, Pharmacal Research Labs, Inc.) between animals. After each trial, mice were dried with paper towels and returned to home cages placed on a heating pad. On day 1, a single swim trial of 15 min was conducted. Immobility was defined as stationary posture for at least 2 s with only minor movements to keep the head above water (McLaughlin et al., 2003; Castagne et al., 2011). On day 1, immobility was quantified across the entire 15 min test. For California mouse experiments, immobility was rescored in 5 min bins. On day 2, each mouse was tested in a series of four 6 min swim trials each separated by a 6–7 min return to home cage (McLaughlin et al., 2003; Bruchas et al., 2007a; Land et al., 2008; Carey et al., 2009). The experimenters’ scoring immobility was blind to the treatment groups. Some California mice had difficulty swimming or staying afloat (**Table 1**). Out of 80 California mice tested, five were removed from day 1 testing (6.3%). On day 2, eight mice were removed due to difficulty swimming (10%). These mice were excluded from statistical analysis, and no one treatment group was over-represented (χ^2^, *p* > 0.2). All C57Bl/6J mice completed all trials on days 1 and 2. Following the last swim trial, mice were anesthetized with isoflurane and euthanized by decapitation. Brains were immediately collected, flash frozen on dry ice, and stored at -40°C.

**Table 1:** California mice excluded from analysis due to difficulty in swimming or staying afloat.

Sex	Stress	Trt	Total mice	#Failed day 1	#Failed day 2	# Total fail	%Total fail
Female	Control	Veh	13	2	1	3	23.1
Female	Control	norBNI	9	1	0	1	11.1
Female	Control	U50	10	1	1	2	20.0
Female	Stress	Veh	7	0	1	1	14.3
Female	Stress	norBNI	10	1	0	1	10.0
Female	Stress	U50	10	0	2	2	20.0
Male	Control	Veh	11	0	1	1	9.1
Male	Control	norBNI	10	0	2	2	20.0
Total			80	5	8	13	16.3


*norBNI, nor-binaltophimine.*

### Liquid Chromatography–Mass Spectrometry

Brain and plasma samples were thawed on ice. Brains were transferred to a vial containing 2.8 mm stainless steel grinding balls (OPS Diagnostics, Lebanon, NJ, United States). Brains were homogenized in water at a ratio of 3:1 (vol:wt) using a GenoGrinder 2010 (SPEX SamplePrep, Metuchen, NJ, United States) at 1750 rpm for 30 s × 2; 50 μl aliquots of brain homogenate or plasma were added to a Waters (Milford, MA, United States) 700 μl UPLC 96-well plate and mixed with 150 μl of methanol containing 0.1% formic acid and 10 ml of internal standard (Buspirone, 100 ng/ml). The plate was sealed and mixed for 30 s and centrifuged (Beckman Coulter Allegra X-15R, Pasadena, CA, United States) at 4000 rpm for 10 min; 150 μl of the supernatant was removed and transferred to a new 96-well plate and mixed with 50 μl of 0.1% formic acid in water. Standards were prepared in blank rat plasma or blank rat brain homogenate as indicated above with the addition of norBNI or JDTic. Standards ranged from 1 to 5000 ng/ml for norBNI and 1 to 2000 ng/ml for JDTic in plasma or brain homogenate.

Sample analysis was conducted using an Applied Biosystems API 5000 triple quadrupole mass spectrometer (Foster City, CA, United States) interfaced with a Waters Acquity UPLC System (Milford, MA, United States). Chromatography of norBNI was accomplished using a Phenomenex Luna C18 column (50 mm × 2.0 mm i.d., 5 μm particle size) fitted with a C18 guard cartridge (Torrance, CA, United States). Injection volumes were 10 μl. Two mobile phase solutions were used: (A) was 0.1% formic acid in water and (B) was 0.1% formic acid in acetonitrile. The elution program consisted of a linear gradient starting at 98% (A) and at 1 min following injection progressing to 95% (B) over 4 min and then returning to initial conditions with a flow rate of 500 μl/min. The analytes and internal standard were monitored using multiple reaction monitoring (MRM) in positive ion mode. Multiple MRMs (*m/z* 662.4 → 547.2, 644.2, 98.1, and *m/z* 590.3 → 493.1, 226.1) were summed to increase intensity (Patkar et al., 2013). Chromatography for JDTic was accomplished using a Phenomenex Luna C8 column (150 mm × 4.6 mm i.d., 5 μm particle size) fitted with a C8 guard cartridge (Torrance, CA, United States). Injection volumes were 10 μl. Two mobile phase solutions were used: (A) was 0.5% formic acid in water with 5 mM ammonium acetate and (B) was 0.5% formic acid in 85:15 acetonitrile:water with 5 mM ammonium acetate. The elution program consisted of a linear gradient starting at 95% (A) and at 1 min following injection progressing to 95% (B) over 4 min and then returning to initial conditions with a flow rate of 750 μl/min. The MRM monitored for JDTic was (*m/z* 466.3 → 148.0). NorBNI and JDTic concentrations were determined by comparing the norBNI or JDTic peak area to buspirone (internal standard) peak area ratios in the samples to the ratios found in prepared standards. Pharmacokinetic analysis was conducted with Phoenix WinNonlin version 6.3 (Certara, Princeton, NJ, United States). A non-compartmental model was used to analyze mean data for each time point in brain and plasma.

### Western Blot

First, we validated the phospho-specificity of the pJNK primary antibody (#9255, Cell Signaling, Danvers, MA, United States) (**Figure 4E**). Punch samples from California mice were pooled from four different punches: anterior bed nucleus of the stria terminalis (BNST), posterior BNST, prefrontal cortex, and paraventricular nucleus. Samples were homogenized in ice cold lambda phosphatase buffer (New England Biolabs, Ipswich, MA, United States, 7.5 pH, 50 mM HEPES, 100 mM NaCl, 2 mM DTT, 0.01% Brij 35). After samples were homogenized, 1 mM MnCl2 was added to activate the » phosphatase. Next, calf intestinal alkaline phosphatase (CIP, New England Biolabs, Ipswich, MA, United States) was added at a concentration of 2 units CIP per mg of protein and lambda protein phosphatase (λ PP, New England Biolabs, Ipswich, MA, United States) was added at a concentration of 400 units per ug of protein. An equivalent volume of the lambda phosphatase buffer, but no λ PP or CIP, was added to the control sample. Samples were incubated for 1 h at 37°C. Laemmli buffer (Sigma, St. Louis, MO, United States) was added to the homogenate at a 1:1 dilution and samples were placed on a shaker at 4°C for 1 h. Proteins were denatured at 98°C for 5 min, chilled on ice for 5 min, and separated with gel electrophoresis (12% precast polyacrylamide gel, Bio-Rad). Proteins were transferred to a polyvinylidine fluoride (PVDF) membrane (Bio-Rad, Hercules, CA, United States), rinsed, and blocked with 5% skim milk in 0.1% Triton-X with tris buffered saline (TBS-T). Membranes were incubated overnight in primary antibody (pJNK, #9255, Cell Signaling, 1:500) at 4°C. Membranes were rinsed three times for 5 min in TBS-T and then incubated in secondary antibody (peroxidase conjugated anti mouse, # X0328, Vector) in 5% skim milk in TBS-T for 1 h at room temperature. Following TBS-T washes, developing solution was applied (Bio-Rad), and the blot was imaged on a Bio-Rad ChemiDoc. Membranes were stripped by placing in Restore^TM^ Western Blot Stripping Buffer (Thermo Fisher, Waltham, MA, United States) for 5 min at room temperature. Membranes were washed, blocked for 1 h at room temperature, and incubated overnight in total JNK primary antibody (phospho and non-phospho, #9252, Cell Signaling, 1:1000) at 4°C. Membranes were washed in TBS-T and incubated in peroxidase conjugated antirabbit secondary antibody (#X0126, Vector, 1:2000) for 1 h at room temperature. Following TBS-T washes, developing solution was applied (Bio-Rad), and the blot was imaged on a Bio-Rad ChemiDoc.

Experimental punch samples of the NAc from California mice (Experiments 1 and 2) and C57Bl6 (Experiment 3) treated with 10 mg/kg norBNI or vehicle were homogenized in ice cold buffer (7.4 pH, 20% glycerol, 0.4 M NaCl, 20 mM HEPES, 5 mM MgCl_2_, 0.5 mM EDTA in H_2_O) with protease inhibitor (1% PMSF in EtOH) and phosphatase inhibitor (50 mM NaF). Protein content was measured (Pierce 600 nm Protein Assay) and samples with containing less than 0.1 mg/mL of total protein were excluded from the analysis. Laemmli buffer (Sigma, St. Louis, MO, United States) was added to the homogenate at a 1:1 dilution and samples were placed on a shaker at 4°C for 1 h. Proteins were denatured at 98°C for 5 min, chilled on ice for 5 min, and separated with gel electrophoresis (12% precast polyacrylamide gel, Bio-Rad). Protein was transferred to PVDF membranes (Bio-Rad, Hercules, CA, United States), blocked, and placed in primary pJNK antibody as described above. Secondary antibody incubation and imaging was performed as described above. Each membrane was then stripped and processed for JNK (#9252, Cell Signaling, Danvers, MA, United States, 1:1000) followed by β-Actin (#4970 Cell Signaling, Danvers, MA, United States, 1:2000). All membranes were reimaged after stripping to confirm a lack of signal. pJNK and JNK protein bands were normalized to β-Actin.

### Statistical Analyses

For experiments using the forced swim test (Experiments 1–3, 5, and 6), day 2 immobility data were analyzed using repeated measures ANOVA. All data were square root transformed for analysis to normalize variance between treatment groups. In Experiment 2, social defeat stress was included as an independent variable. For experiments in which there was a main effect of drug treatment, but no drug by trial interaction detected (Experiments 2 and 3), we used planned comparisons to compare drug treatments with vehicle. Pharmacokinetic and western blot data were analyzed with two-way ANOVA analysis.

## Results

### Experiment 1: Effects of norBNI and U50,488 on Immobility in Female California Mice

First, we investigated the effects of norBNI and the KOR agonist U50,488 on immobility in female California mice. On day 2, repeated measures ANOVA showed a trend for the effects of KOR ligands to have different effects in control and stressed mice (**Figure 1C**; drug × stress × trial interaction; *F*_6,141_ = 1.77, *p* = 0.09). In control females, there was a significant main effect of drug treatment (**Figure 1C**; drug; *F*_2,25_ = 3.42, *p* = 0.049) but no drug by trial interaction. Females treated with U50,488 spent more time immobile than vehicle (planned comparison, *p* < 0.05), whereas norBNI-treated females did not differ from vehicle (**Figure 1C**). In stressed females, there was no main effect of drug treatment and no drug by trial interaction (all *p*’s > 0.42). All groups showed increased immobility between the first and fourth trials (**Figure 1B**, paired *t*-tests, all *p*’s < 0.05). On day 1 of testing, there were no differences in total immobility or within any of the 5 min bins analyzed (**Table 2**; all *p*’s > 0.27). The most important finding in these analyses is that norBNI did not reduce immobility in either control or stressed female California mice.

**Table 2:** Mean ± SEM immobility (sec) data from Day 1 for female and male California mice.

Time point	Control vehicle	Control norBNI	Control U50,488	Stress vehicle	Stress norBNI	Stress U50,488
**Female California mice**	
First 5 min (Bin 1)	31.8 ± 6.5	36.6 ± 8.7	27.6 ± 8.7	21.4 ± 11.2	22.5 ± 10.7	21.0 ± 8.3
Second 5 min (Bin 2)	48.6 ± 11.2	53.5 ± 8.1	41.7 ± 9.1	55.17 ± 13.3	35.5 ± 38.6	56.6 ± 13.6
Third 5 min (Bin 3)	63.9 ± 15.2	66.9 ± 13.1	46.0 ± 8.7	66.7 ± 16.9	34.5 ± 9.9	51.6 ± 19.1

**Time point**	**Vehicle**	**norBNI**				

**Male California mice**	
First 5 min (Bin 1)	27.9 ± 5.4	28.5 ± 6.5				
Second 5 min (Bin 2)	57.1 ± 8.1	40.3 ± 8.2				
Third 5 min (Bin 3)	73.0 ± 11.8	42.5 ± 6.9^∗^				


*Immobility scores analyzed with two-way ANOVA (stress and drug treatment). ^∗^*p* < 0.05 vs. corresponding vehicle.*

### Experiment 2: Effects of norBNI on Immobility in Male California Mice

**FIGURE 2:**
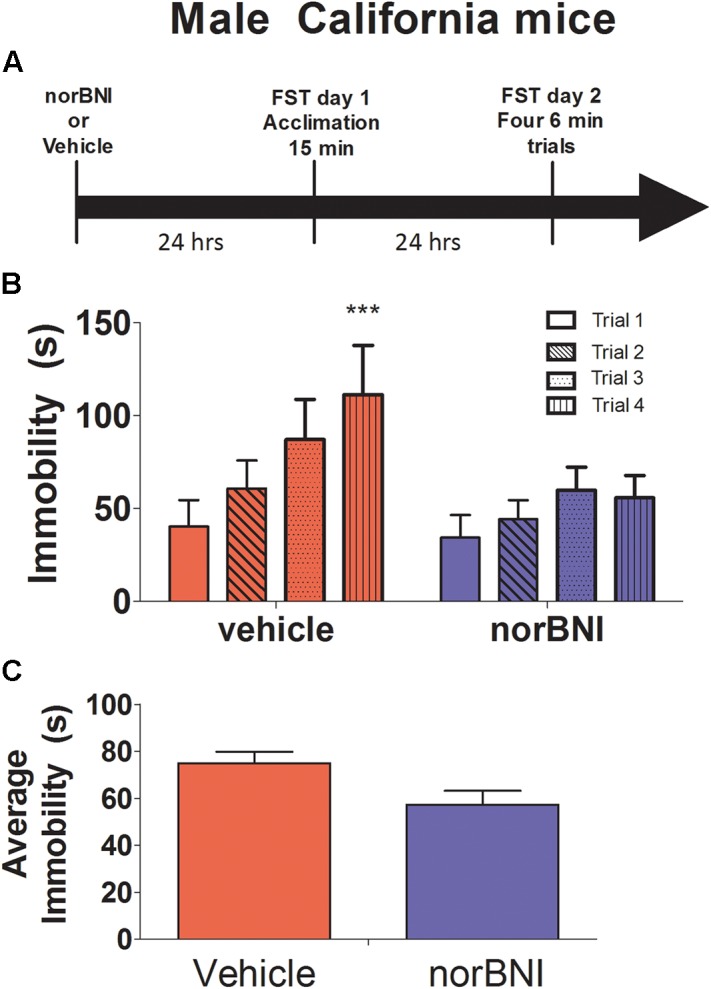
Effects of 10 mg/kg norBNI on immobility in male California mice. **(A)** Experimental timeline of analyses. **(B)** Day 2 immobility for the four trials for male California mice. **(C)** Average immobility across the four Day 2 trials. Immobility scores were analyzed with one-way repeated measures ANOVA testing for drug treatment. ^∗∗∗^*p* < 0.001, ^∗^*p* < 0.05, repeated measures ANOVA of four trials, *n* = 11–10 per group. Error bars are SEM.

In male California mice, norBNI treatment blocked increases in immobility across the four day 2 trials (**Figures 2A–C** drug × trial interaction; *F*_3,57_ = 3.69, *p* = 0.02). There was no difference in immobility between vehicle and norBNI treated males on day 1. However, on day 2, immobility increased almost threefold in vehicle treated males (**Figure 2B** paired *t*_10_ = 5.9, *p* < 0.001) and by less than twofold in males treated with norBNI (**Figure 2B** paired *t*_9_ = 2.4, *p* = 0.04). Similar results were observed on day 1, as norBNI blunted increases in immobility across the 5 min bins of the single 15 min swim session (**Table 2**, *F*_2,38_ = 3.98, *p* = 0.03). These results suggest that the effects of norBNI are similar in male California mice as in other rodents.

### Experiment 3: Effects of norBNI on Immobility in Male and Female C57Bl/6J

**FIGURE 3:**
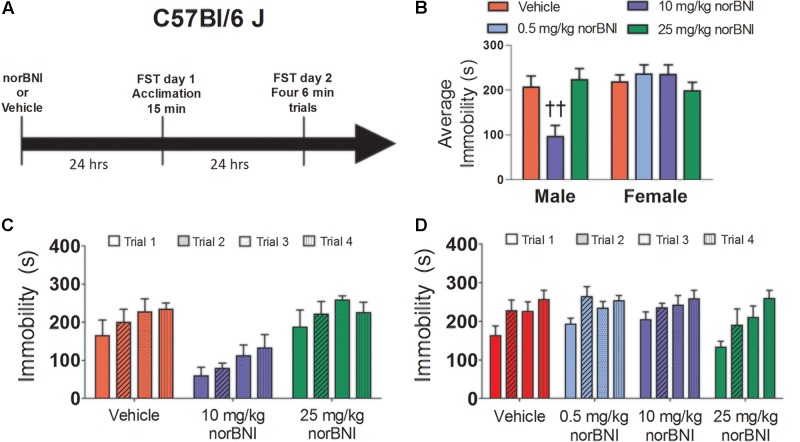
Effects of norBNI on immobility in male and female C57Bl/6J mice. **(A)** Experimental timeline. **(B)** Average immobility across the four Day 2 trials for male and female C57Bl/6J mice. Immobility scores were analyzed with one-way repeated measures ANOVA testing for drug treatment. ††*p* < 0.01 compared to vehicle (planned comparison of marginal means). Day 2 immobility for the four trials for male **(C)** and female **(D)** mice. For males, *n* = 5 per group, and for females, *n* = 6–10 per group. Error bars are SEM.

In male C57Bl/6J, repeated measures ANOVA indicated that norBNI dose-dependently reduced immobility (**Figure 3B**; drug; *F*_2,12_ = 7.59, *p* < 0.01). Compared to vehicle, males treated with 10 mg/kg of norBNI (planned comparison; *p* < 0.01), but not 25 mg/kg had significantly lower immobility. As in previous reports, 10 mg/kg norBNI reduced immobility across all four trials (**Figure 3C**). In contrast, in female C57Bl/6J, there was no effect of drug treatment (**Figure 3B**; drug; *F*_3,25_ = 0.79, *p* = 0.51) and no trial by treatment interaction (**Figure 3D**, drug × trial; *F*_9,75_ = 0.73, *p* = 0.68). On day 1, the 25 mg/kg dose increased immobility in males (**Table 3**; planned comparison; *p* = 0.02), whereas no differences were observed in females. Overall, our results in C57Bl/6J mice replicate the finding from California mice that 10 mg/kg norBNI decreases immobility in males but not females.

**Table 3:** Mean ± SEM total immobility (sec) data from Day 1 for female and male C57Bl/6J.

Sex	Vehicle	0.5 mg/kg norBNI	10 mg/kg norBNI	25 mg/kg norBNI
Male	245.8 ± 76.0		196.8 ± 64.0	505 ± 67.1^∗^
Female	280.1 ± 43.0	334.2 ± 56.7	370.3 ± 74.7	415.5 ± 64.6


*Data analyzed with one-way ANOVA. ^∗^*p* < 0.05 vs. corresponding vehicle.*

### Experiment 4: Pharmacokinetics of norBNI and JDTic and Quantification of pJNK in the Nucleus Accumbens of Male and Female C57Bl/6J

**FIGURE 4:**
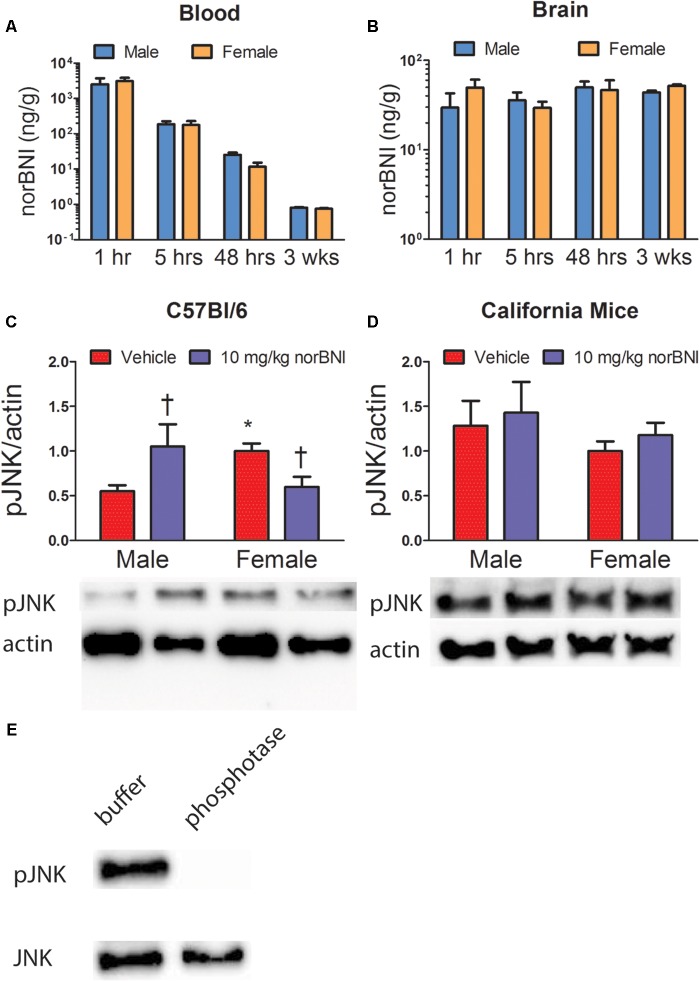
Pharmacokinetics of norBNI in plasma **(A)** and whole brain **(B)** of male and female C57Bl/6J mice treated with 10 mg/kg norBNI, *n* = 4 per group. **(C,D)** Phosphorylation of c-Jun N-terminal kinase protein relative to β-Actin protein in the NAc normalized to vehicle treated females. Effects of norBNI on phosphorylated JNK in male and female C57Bl/6 **(C)** and California mice **(D)** in nucleus accumbens punch samples, *n* = 5–9 per group. **(E)** Validation of phospho-specific JNK antibody using calf intestinal phosphatase and λ phosphatases. All data were analyzed with two way-ANOVA. Error bars are SEM. ^†^*p* < 0.05 vs. vehicle (planned comparison). ^∗^*p* < 0.05 vs. vehicle male (planned comparison).

We next tested whether the lack of efficacy of norBNI in females could be explained by differences in metabolism or diffusion into the brain (**Figure 4** and **Table 4**). There were no significant sex differences in the amount of norBNI in plasma at any time point (**Figure 4A**; sex × time interaction; *F*_3,24_ = 0.71, *p* = 0.56) or in the brain at any time point (**Figure 4B**; sex × time interaction; *F*_3,24_ = 0.65, *p* = 0.59). Levels of norBNI in the plasma decreased over time at a similar rate in males and females (**Figure 4B**; main effect of time; *F*_3,24_ = 6.54, *p* < 0.01). Interestingly, norBNI levels did not decrease over time in the brain (**Figure 4A**). Overall, these data indicate that there are no sex differences in the pharmacokinetics of norBNI in the brain or plasma.

**Table 4:** Pharmacokinetic constants for 10 mg/kg norBNI administered to male and female C57Bl/6J.

	Plasma		Brain	
		
	Male	Female	Male	Female
*K* (1/h)	8.38E-03	6.03E-03	1.23E-04	4.00E-04
*t*1/2 (h)	8.30E+01	1.15E+02	5.63E+03	1.73E+03
Cmax (ng/mL)	2.52E+03	3.11E+03	5.92E+01	6.23E+01
Clast (ng/mL	8.07E-01	7.51E-01	4.37E+01	5.19E+01
AUC (h^∗^ng/mL)	1.44E+04	1.78E+04	2.33E+04	2.86E+04


*Data analyzed with two-way ANOVA.*

Analysis of variance suggested that males have higher levels of JDTic in the brain than females (Supplementary Figure S1A; main effect; *F*_1,24_ = 5.57, *p* = 0.03). However, Levene’s test indicated there was heterogeneity of variance (*p* < 0.001). Non-parametric analyses indicated that 48 h post-injection males had higher levels of JDTic in brain than females (Mann–Whitney; *U* = 0.01, *p* = 0.03). There were no sex differences at any other time point (all *p*’s > 0.11). In plasma, ANOVA analyses did not detect sex differences in JDTic (Supplementary Figures S1B,C; main effect of sex; *F*_1,24_ = 2.65, *p* = 0.12) or in the rate of change (Supplementary Figure S1B; sex × time interaction; F_3,24_ = 1.17, *p* = 0.34). Levels of JDTic in plasma decreased over time (Supplementary Figure S1B; main effect of time; *F*_3,24_ = 39.24, *p* < 0.0001). Overall, these data suggest that in the whole brain, JDTic levels were lower 48 h after injection but not after 3 weeks.

Next we examined the effects of norBNI on phosphorylated JNK, thought to be an important pathway mediating norBNI action. In C57Bl/6J mice, the effects of norBNI were different in NAc punch samples from males and females (**Figure 4C**, *F*_1,18_ = 7.79, *p* = 0.01). Males treated with norBNI had increased pJNK expression in the NAc compared to vehicle (*p* < 0.05). Interestingly, vehicle treated females had increased pJNK expression compared to vehicle treated males (*p* < 0.05), and females treated with norBNI had lower expression of pJNK compared to vehicle treated females (*p* < 0.05). These results indicate there are important sex differences in JNK phosphorylation in the NAc of C57Bl/6 mice. We conducted similar analyses in California mice but observed no differences in pJNK expression (**Figure 4D**, all *p*’s > 0.35). There were also no differences in the amount of total JNK in either California mice or C57Bl/6J.

## Discussion

The inhibitory effects of the KOR antagonist norBNI on immobility in the forced swim test are well-established in male rodents. In male C57BL/6J and California mice, we obtained results that are similar to these previously described effects of norBNI. Yet, in both species, we did not observe similar results in females, even though several doses were examined. Pharmacokinetic analyses demonstrated that norBNI concentrations in the brain were similar in males and females, suggesting that the mechanism of this sex difference is not driven by differences in the blood brain barrier or metabolism. Although there were sex differences in the effects of norBNI on phosphorylated JNK in C57Bl/6J, this pattern did not generalize to California mice. Together, these results suggest that immobility in the forced swim test is less dependent on KOR activation in females than males.

Consistent with previous studies (Pliakas et al., 2001; Mague et al., 2003; Beardsley et al., 2005; Falcon et al., 2015), male California mice and C57Bl/6J mice treated with norBNI had lower immobility levels than vehicle treated mice. In both species, mean time spent immobile in vehicle treated mice was similar in males and females, as observed in Long Evans rats (Colom-Lapetina et al., 2017). Unexpectedly, none of the doses of norBNI we tested reduced immobility in females of either species. We conducted detailed analyses of behavior on both days 1 and 2. Consistently, norBNI failed to reduce immobility in females of the two species. Several studies have reported inverted U-shaped functions for norBNI (Todtenkopf et al., 2004; Knoll et al., 2007), suggesting that at higher doses, norBNI may have non-specific effects. For example, we observed that 10 mg/kg but not 25 mg/kg of norBNI reduced immobility in male C57Bl6/J mice. In female C57Bl6/J, we included a lower dose of norBNI based on previous work in female rodents showing that lower doses of antidepressants have been found to have greater efficacy than higher doses in the forced swim (Kokras et al., 2015) and social interaction (Greenberg et al., 2014) tests. Our results suggest the lack of norBNI efficacy in females is not a result of dosage insensitivity. An alternate possibility is that immobility in the forced swim test is not sensitive to any form of antidepressant in female California mice and C57Bl/6J. In mice, there is clearly variability across strains in how different types of antidepressants affect immobility, with male C57Bl6/J showing significant decreases in immobility to desipramine and paroxetine but not fluoxetine (Pedersen et al., 1994; Ford and Erlinger, 2004). Importantly, the tricyclic antidepressant amitriptyline reduced immobility in female C57Bl/6J mice (Bosch and Neumann, 2012). This suggests that female C57Bl/6J respond to at least one form of standard antidepressant in the forced swim test.

Several mechanisms might explain sex differences in the effects of KOR ligands on immobility. First, several reports have indicated that sex differences in pharmacokinetics can have important influences on drug effects (Soldin and Mattison, 2009; Greenblatt et al., 2014). There were no differences between males and females in the levels of norBNI in the brain or plasma at any of the time points measured. Interestingly, while norBNI was slowly eliminated from plasma over several days, there was no decrease in brain levels of norBNI. This result aligns with previous findings showing that norBNI remains in the brains of male mice over 21 days after single injection (Patkar et al., 2013). Our data show that this finding extends to females. Second, we tested whether there were sex differences in the pJNK. Previous work showed that norBNI increased pJNK in striatum (Bruchas et al., 2007b), and we focused our analyses on the NAc based on the evidence that forced swim induces the expression of immediate early genes in the NAc (Carlezon et al., 2005; Kreibich and Blendy, 2005). Consistent with previous work, norBNI increased pJNK expression in male C57Bl/6J. In contrast, norBNI decreased pJNK in female C57Bl/6J although control females also had higher pJNK expression than control males. This sex difference in pJNK could be mediated by higher corticosterone levels in female C57Bl/6J (Goel and Bale, 2008), as corticosterone facilitates pJNK (Di and Tasker, 2004; Qi et al., 2005). While it is possible that sex differences in norBNI regulation of JNK contribute to sex differences in norBNI regulation of behavior, there were no effects of norBNI on pJNK in California mice. It is possible that KOR effects on immobility occur outside the NAc, perhaps in the dorsal striatum. In guinea pigs, autoradiography showed that males have increased KORs in the caudate putamen compared to females (Wang et al., 2011). If this increased expression of KOR in males is conserved, it could contribute to greater male responsiveness to norBNI in forced swim tests. Finally, immobility in females may be less dependent on endogenous KOR signaling. Reduced sensitivity to KOR in females has been reported in studies of using intra-cranial self-stimulation (Russell et al., 2014).

We did not observe increased immobility in females exposed to social defeat. Social defeat increased immobility in adolescent male mice (Iñiguez et al., 2014) but decreased immobility in male rats (Der-Avakian et al., 2014). In these studies, the forced swim test was performed within 24 h of the last episode of defeat, so it’s possible that we may have seen stronger effects of defeat on immobility if we had conducted the forced swim test within 1 day after the last episode of defeat. Alternatively, the inconsistent effect of defeat on immobility supports the assertion that immobility in the forced swim test may be more of a measure of alternative coping strategies rather than a depression-like behavior (Molendijk and de Kloet, 2015; Colom-Lapetina et al., 2017). There was some evidence that California mice may use immobility to a greater extent than other rodents. In general, mean levels of immobility in California mice were similar to a recent report (Hyer and Glasper, 2017) and higher than C57Bl/6J. Higher immobility times in California mice could be due to either higher body mass (mean ± SEM; California mouse male: 34.3 ± 1.5 g female: 38.3 ± 1.0 g; C57Bl/6J male: 24.4 ± 0.3 g, female: 19.9 ± 0.3 g) or due to species differences in habitats. California mice have evolved to live in habitats with small streams with little need for swimming (Ribble, 1992), whereas house mice (from which C57Bl/6J were derived) are thought to use swimming as a means for colonizing new habitats (Pocock et al., 2005).

Overall, our data suggest that immobility in the forced swim test is less dependent on KOR in female rodents compared to males. The mechanism by which this sex difference occurs is still unclear, although our data indicate that sex differences in the pharmacokinetics of norBNI or the level of pJNK in the NAc are unlikely mechanisms. While the forced swim test has been useful for identifying pharmaceuticals with antidepressant properties (Molendijk and de Kloet, 2015), the mechanisms mediating the effects of antidepressants on immobility are likely different from those that result in clinical responses. For example, selective serotonin reuptake inhibitors (SSRIs) act acutely to reduce immobility while chronic SSRI treatment is needed for clinical responses. It will be important to test whether there are sex differences in KOR antagonists in other stress models such as social defeat or chronic mild stress. For example, defeat-induced social avoidance can be reversed by chronic but not acute treatment with SSRI in both males (Berton et al., 2006) and females (Greenberg et al., 2014). Recent work suggests that in the context of social defeat, KOR antagonists have greater efficacy if administered before social defeat (Donahue et al., 2015) rather than after defeat (Browne et al., 2017). Overall, our results suggest that a 2-day forced swim test may not be an optimal test for assessing the therapeutic potential of novel KOR ligands in females, and emphasize the need to study both sexes in preclinical studies.

## Author Contributions

AL-M, FC, TF, JS, and BT designed the research; AL-M, AW, MZ, VM, SR-M, RH, EOS, TC, AL, and RS collected the data; AL-M and BT analyzed the data; AL-M, JS, RS, TF, and BT wrote the paper.

## Conflict of Interest Statement

The authors declare that the research was conducted in the absence of any commercial or financial relationships that could be construed as a potential conflict of interest.
