# Supplementary material for: Sex Differences in the Effects of a Kappa Opioid Receptor Antagonist in the Forced Swim Test
Source: Front Pharmacol. 2018 Feb 14;9:93. doi: 10.3389/fphar.2018.00093 (PMC5817081; doi:10.3389/fphar.2018.00093)
Supplement: Supplementary file 1 [file Image_1.pdf]

Figure S1: Pharmacokinetic data for JDTic in male and female C57Bl/6J mice. Plasma (A) and brain (B) concentrations of JDTic at different time points including pharmacokinetic constants (C). * $p < 0.05$, effect of sex.

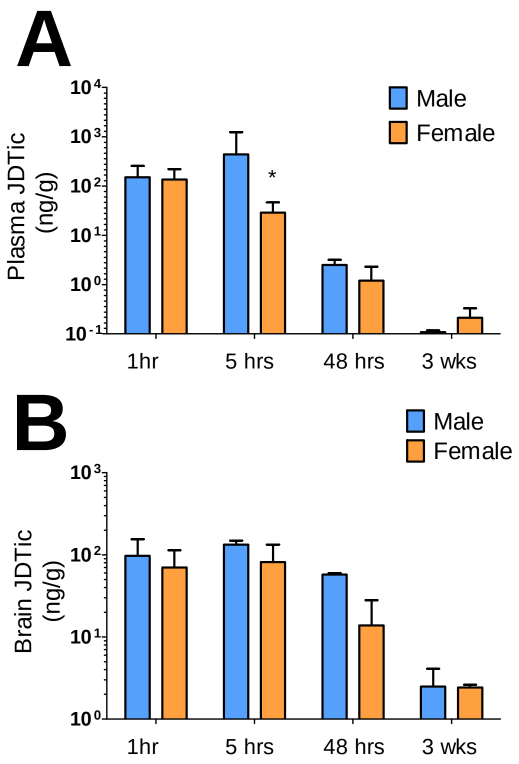

C

	Brain		Plasma	
	**Male**	**Female**	**Male**	**Female**
**K (1/h)**	0.00744	0.003808	0.006874	0.003823
**t1/2 (h)**	93.2	182	100.8	181.3
**Cmax (ng/ml)**	133.75	81.795	437.95	135.7
**Clast (ng/ml)**	2.508	2.425	0.109	0.212
**AUC (h*ng/ml)**	18329	6086	11315	1369
